# Hypersensitivity reaction and acute immune-mediated thrombocytopenia from oxaliplatin: two case reports and a review of the literature

**DOI:** 10.1186/1756-8722-3-12

**Published:** 2010-03-26

**Authors:** Marnelli A Bautista, W Tait Stevens, Chien-Shing Chen, Brian R Curtis, Richard H Aster, Chung-Tsen Hsueh

**Affiliations:** 1 Department of Pathology and Laboratory Medicine, Loma Linda University Medical Center, Loma Linda, CA 92354, USA; 2 Division of Medical Oncology and Hematology, Loma Linda University Medical Center, Loma Linda, CA 92354, USA; 3 Platelet and Neutrophil Immunology Laboratory, BloodCenter of Wisconsin, Milwaukee, WI 53233, USA; 4 Department of Medicine and Pathology, Medical College of Wisconsin, Milwaukee, WI 53226, USA

## Abstract

**Background:**

Oxaliplatin is a platinum compound used in the treatment of gastrointestinal malignancies, including colorectal cancer. The incidence of hypersensitivity reaction in patients receiving oxaliplatin is approximately 15%, with severe reaction (grade 3 and 4) occurring in 2% of patients.

**Case presentation:**

We report two patients with metastatic colorectal cancer who developed *de novo *hypersensitivity reaction and acute thrombocytopenia after oxaliplatin infusion. Both patients had oxaliplatin treatment several years before and exhibited hypersensitivity on the third dose of oxaliplatin in recent treatment. Oxaliplatin was discontinued when clinical reaction was identified. Both patients were confirmed to have strong oxaliplatin-induced IgG platelet-reactive antibodies. Both patients' thrombocytopenia resolved within two weeks after discontinuation of oxaliplatin. One patient had disease stabilization lasting for three months without chemotherapy. Both patients subsequently received other chemotherapeutic agents without evidence of hypersensitivity reaction or immune-mediated thrombocytopenia.

**Conclusion:**

We recommend vigilant monitoring of complete blood count and signs and symptoms of bleeding after the occurrence of oxaliplatin-induced hypersensitivity to avoid serious complications of immune-mediated thrombocytopenia.

## Background

Oxaliplatin is a third-generation platinum derivative that has been widely used in patients with gastrointestinal malignancies including colorectal cancer (CRC). The combination of 5-fluorouracil, leucovorin and oxaliplatin (FOLFOX) has been demonstrated in several studies to increase survival rate and reduce the risk of disease progression in patients with metastatic CRC and stage III colon cancer [1,2]. Thrombocytopenia has been noted in more than 70% of patients receiving FOLFOX., and is usually self-limited and related to myelosuppression from oxaliplatin [2]. The isolated and acute decline in platelets after FOLFOX treatment is thought to be immune-mediated, and is referred as drug-induced immune thrombocytopenia (DIIT). Oxaliplatin-dependent antibody against platelet glycoprotein IIb/IIIa complex has been identified in patients with oxaliplatin-induced immune thrombocytopenia [3].

The hypersensitivity reaction associated with oxaliplatin typically consists of rigors, fever, rash, tachycardia, and dyspnea. The incidence in patients with CRC was reported as high as 15% and mainly occurred shortly after infusion in patients who had prior exposure to oxaliplatin [4,5]. The mild hypersensitivity reaction (grade 1 or 2) usually responds to discontinuation of oxaliplatin and supportive treatment with antihistamine agents and steroid. Frequently, patients with mild hypersensitivity reaction can be re-treated with oxaliplatin by adding appropriate pre-medications such as antihistamine agents and steroid, and increasing infusion time with more diluted concentration [5,6]. Severe and potentially fatal hypersensitivity reaction with symptomatic bronchospasm, angioedema, hypotension and anaphylaxis, occurred in about 2% of patients receiving oxaliplatin treatment [7,8]. Although the manufacturer recommends not to re-treat with oxaliplatin after the incidence of severe hypersensitivity reaction, a desensitization protocol has been successfully implemented in patients with grade 3 hypersensitivity [9].

Here, we describe two cases of acute thrombocytopenia with concurrent oxaliplatin-induced hypersensitivity reaction in patients with metastatic CRC. Both patients had prior oxaliplatin treatment without occurrence of hypersensitivity, or acute thrombocytopenia and received oxaliplatin several years later due to disease progression with non-responsiveness to other chemotherapeutic regimens.

## Case Presentation

### Case one

A 60-year-old African-American male was diagnosed with stage IV rectosigmoid colon cancer with liver metastasis in 2004. He underwent abdominoperineal resection of rectosigmoid cancer followed by six months of FOLFOX chemotherapy with partial response in liver metastasis. Subsequently, he received pelvic chemoradiation with capecitabine. However, the liver metastasis progressed while waiting for surgical evaluation. He received FOLFOX and bevacizumab, 10 months after the last dose of FOLFOX. After three cycles of treatment, oxaliplatin was replaced by irinotecan because of persistent grade 2 neuropathy. Due to disease progression, he was given additional treatment with bevacizumab, irinotecan and panitumumab with disease stabilization lasting for more than 12 months. Subsequently, radiofrequency ablation of the two hepatic metastatic lesions was performed. He developed congestive heart failure requiring warfarin treatment, and bevacizumab was discontinued.

**Figure 1:**
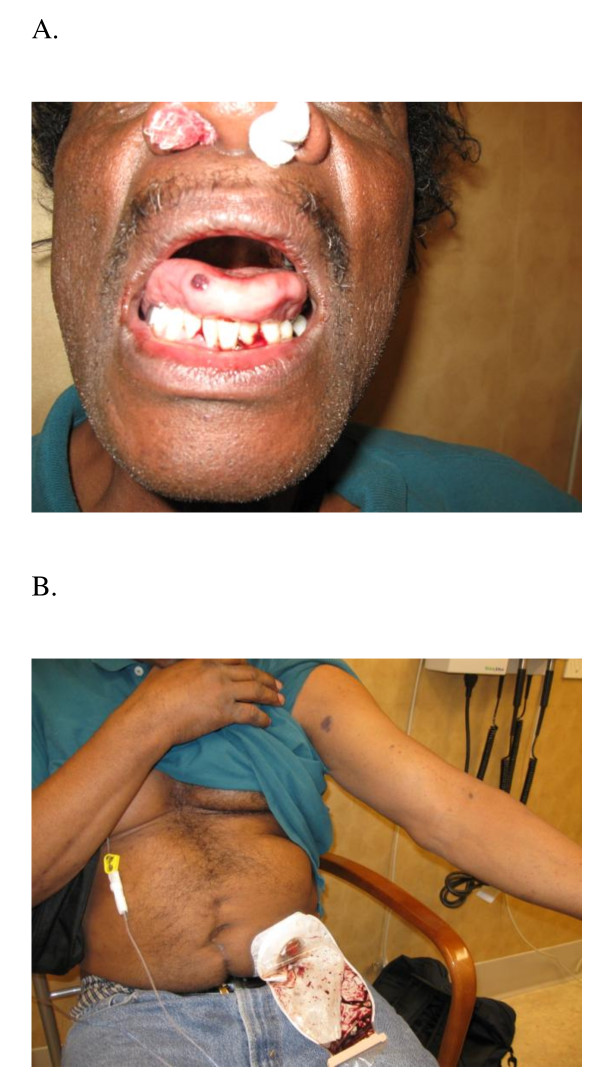
**Photographs form patient one taken 24 hours after oxaliplatin infusion**. A. Intense epistaxis with placement of nasal packing, and tongue-hematoma formation.  B. Upper extremity petechiae, bruises and accumulation of blood in the colostomy.

**Figure 2:**
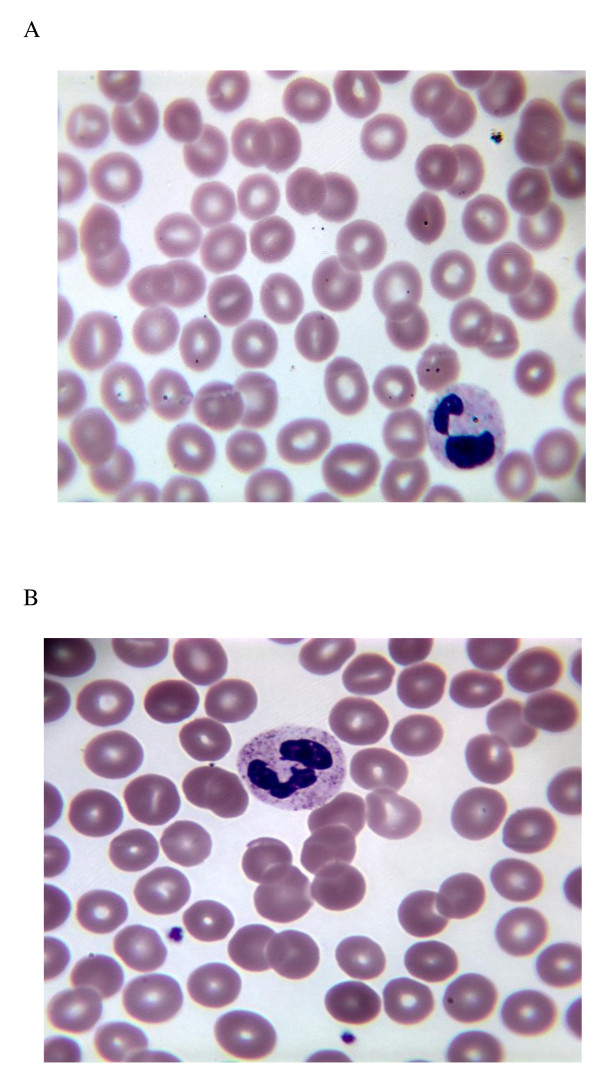
**Peripheral blood smears**. A. Patient one: markedly decreased platelets without significant schistocytosis. (Wright's stain, 1,000×) B. Patient two: moderate decline in platelets. (Wright's stain, 1,000×)

In December 2008, due to disease progression and improvement of neuropathy, he was restarted on dose-reduced FOLFOX, with oxaliplatin 70 mg/m^2 ^plus leucovorin (LV) 400 mg/m^2 ^intravenous infusion over two hours followed by 5-fluorouracil (5-FU) 2400 mg/m^2 ^infusion over 46 hours every two weeks. In mid-January 2009, during the third cycle of chemotherapy, one hour into a planned two-hour infusion of oxaliplatin and LV, he developed hypersensitivity reaction with rigors, chills, bronchospasm and decreased oxygen saturation. Chemotherapy infusion was immediately discontinued. The symptoms resolved after the patient received oxygen supplementation, antihistamine agents and steroid. Infusion of oxaliplatin and LV was resumed and was completed with a slower infusion rate. However, he experienced mild gingival bleeding at the end of infusion and was instructed to return to clinic if the condition worsened. In the evening, he developed epistaxis with persistent gingival bleeding and had bright red blood emanating from the colostomy site. He was found to have prominent skin petechiae, bruises and tongue hematoma the next day (Fig. 1A and 1B). Complete blood count (CBC) showed an abrupt and marked decrease in platelet count from 226 × 10^9^/L (measured one day prior to chemotherapy) to 4 × 10^9^/L about 24 hours after the oxaliplatin infusion. Leukocyte and erythrocyte counts, hemoglobin and hematocrit levels were within the normal reference range. Peripheral blood smear revealed severe decrease in platelets, with minimal schistocytosis. No blasts or atypical cells were observed (Fig. 2A). The prothrombin time was mildly prolonged with an International Normalized Ratio (INR) of 1.9 due to warfarin prophylaxis for congestive heart failure. Warfarin was discontinued to help control hemorrhage. Identification of platelet antibody was performed at the BloodCenter of Wisconsin (Milwaukee, WI) and revealed presence of both non-specific Immunoglobulin G (IgG) and oxaliplatin-induced, IgG platelet antibodies. Additional studies such as human immunodeficiency virus (HIV) testing, hepatitis B and C screening, and antinuclear antibody (ANA) analysis have been performed to exclude other causes of thrombocytopenia. Each yielded a negative result.

**Figure 3:**
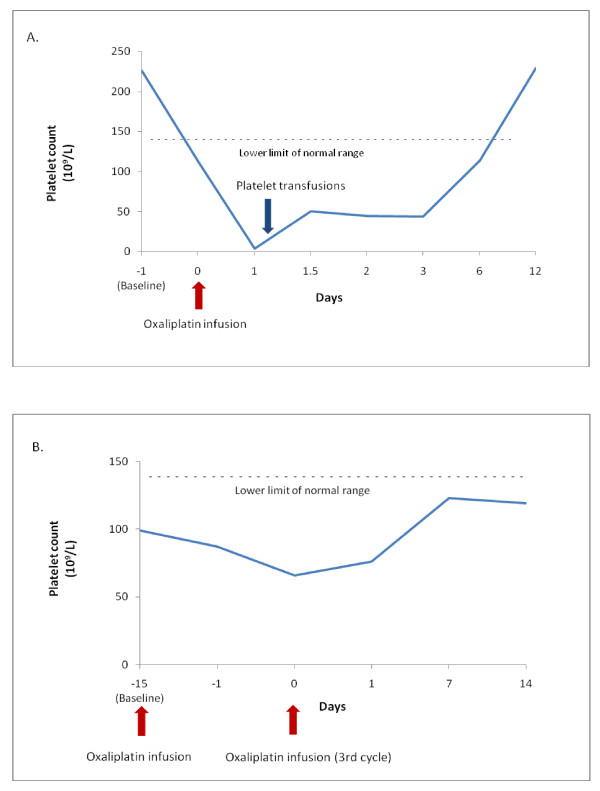
**Plots displaying the trend of platelet counts**. A. Patient one: the steep platelet decline occurring 24 hours after completion of oxaliplatin infusion, with concurrent bleeding diathesis. Two units of apheresis platelets were provided. B: Patient two: moderate decrease in platelet count without evidence of bleeding. Platelet drop was not as drastic as patient one due to discontinuation of the rest of oxaliplatin treatment.

He was admitted for prompt management and received two consecutive apheresis platelet transfusions, and one adult unit of plasma. The platelet count increased to 51 × 10^9^/L with resolution of bleeding overnight. He was sent home after two days of observation without requiring additional platelet infusions. Follow-up CBC measurements showed increasing platelet counts, ultimately reaching a normal level within twelve days after the incident (Fig. 3A). His warfarin treatment was resumed after resolution of thrombocytopenia. His cancer-related symptoms remained stable, with decreasing CEA levels for three months, even without the use of chemotherapy. An abdominal CT scan performed one month after the hypersensitivity reaction indicated stable disease. However, three months after, he experienced mild cancer-related symptom, requiring resumption of chemotherapy with 5-FU and LV. Bevacizumab was later added due to disease progression. He did not experience any hypersensitivity reaction or acute thrombocytopenia with these drugs.

### Case two

A 66-year-old Hispanic female with type 2 diabetes mellitus, degenerative arthritis, and diverticulitis was diagnosed with stage IV colon cancer with lung and liver metastases in 2006 after surgical resection of sigmoid colon cancer. She was treated with first-line chemotherapy including bevacizumab, capecitabine and oxaliplatin from March 2007. Oxaliplatin was discontinued after four months of treatment due to severe neuropathy. At that time, she achieved disease stabilization with gradual decline of CEA levels. She continued to receive bevacizumab and capecitabine for eight months until disease progression. Subsequently, she was enrolled in a clinical study and received cetuximab, bevacizumab and irinotecan for four months with good control of disease. She was withdrawn from the clinical trial due to significant toxicities. Afterwards, she developed progressive disease on single-agent cetuximab.

In January 2009, with improvement of neuropathy secondary to prior oxaliplatin use, chemotherapy with bevacizumab, oxaliplatin and 5-FU every two weeks was initiated. She tolerated the first two cycles without major side effects. In early March 2009, during her third cycle of chemotherapy, she experienced upper body skin rash and pruritis shortly (5-10 minutes) after starting oxaliplatin treatment. Oxaliplatin infusion was discontinued immediately. The hypersensitivity symptoms resolved after steroid and antihistamine treatment. No petechiae or mucosal bleeding was noted. Due to our prior experience with patient one, a CBC was immediately obtained after the onset of hypersensitivity symptoms. A drop in platelet count from 87 × 10^9^/L to 66 × 10^9^/L was noted despite only receiving a small fraction (less than 1/10) of planned oxaliplatin treatment. Her platelet count was 99 × 10^9^/L fifteen days prior to this event (Fig 3B). A peripheral blood smear confirmed moderate decrease in platelets with normal morphology (Fig. 2B). No schistocytes were identified. Oxaliplatin treatment was not resumed because of hypersensitivity reaction with concomitant mild thrombocytopenia; a presentation similar to patient 1. A serum sample for platelet antibody analysis was also sent to the BloodCenter of Wisconsin (Milwaukee, WI), which later demonstrated the presence of IgG oxaliplatin-induced platelet antibody. The other CBC parameters revealed mild decrease in WBC count, with normal hemoglobin and hematocrit levels. Coagulation panel and bilirubin were also within the normal reference range. HIV, hepatitis B and C screening, and ANA analysis were all negative. The possibility of a platelet transfusion was offered and discussed in case of aggravation of the thrombocytopenia. However, the patient declined infusion of blood products due to religious background. Oral prednisone (20 mg, three times a day, for two days) was prescribed. Her subsequent platelet counts gradually increased within two weeks (Fig. 3B). She resumed chemotherapy with bevacizumab, 5-FU and LV without significant thrombocytopenia or hypersensitivity reaction. Nonetheless, she developed progressive disease several weeks later. She was consequently treated with bevacizumab, irinotecan and 5-FU without any evidence of hypersensitivity reaction or acute thrombocytopenia.

## Conclusion

We describe two cases of immune-mediated thrombocytopenia immediately following the onset of the hypersensitivity reaction in patients with metastatic CRC receiving oxaliplatin treatment. To our knowledge, this is the first report demonstrating the association between *de novo *oxaliplatin-induced hypersensitivity reaction and immune-mediated thrombocytopenia from oxaliplatin. Both patients received FOLFOX chemotherapy several years prior without hypersensitivity symptoms or evidence of acute thrombocytopenia. However, both patients experienced hypersensitivity reaction on retreatment with oxaliplatin. In both cases, acute thrombocytopenia transpired immediately after the occurrence of hypersensitivity symptoms. Our first patient developed severe thrombocytopenia with mucocutaneous bleeding after completing the rest of oxaliplatin treatment, and was hospitalized for platelet transfusion and close observation. For our second patient, a Jehovah's Witness, oxaliplatin infusion was not resumed after the occurrence of hypersensitivity reaction with moderate thrombocytopenia. Both patients had oxaliplatin appended to their drug allergy list to prevent re-exposure.

**Figure 4:**
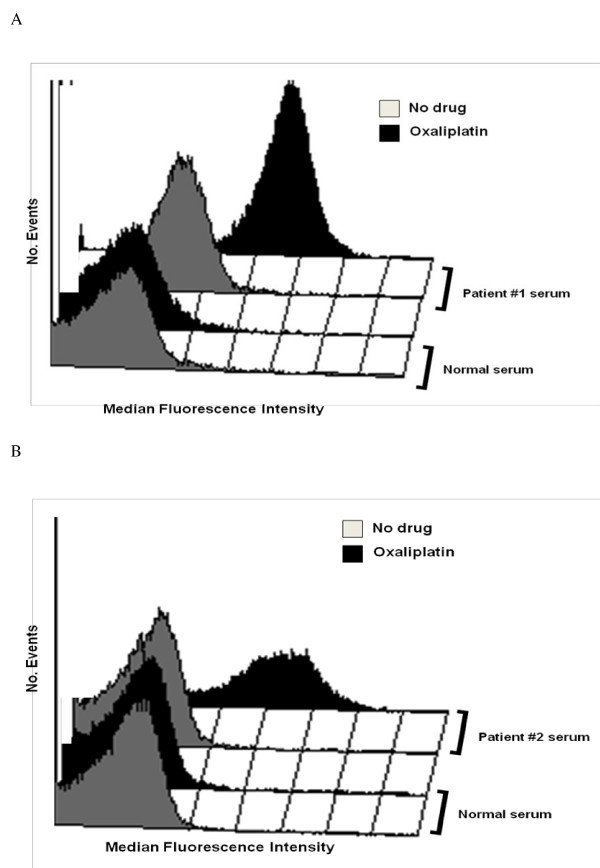
**Detection of oxaliplatin-dependent platelet antibodies**. The assay was performed by flow cytometry in patient one (A) and patient two (B). Both patients had IgG antibodies that reacted strongly with normal platelets in the presence of 0.1 mg/ml oxaliplatin, but not in the absence of drug. No reaction was obtained with normal serum in the presence of drug.

In order to verify that the isolated, precipitous drop in platelet counts was due to oxaliplatin, serum samples from our two patients were collected and sent for drug-induced platelet antibody analyses to the BloodCenter of Wisconsin (Milwaukee, WI). Detection of oxaliplatin-dependent antibodies in patients' sera was performed via flow cytometry [3]. Normal group O platelets were incubated with test serum in the presence and absence of oxaliplatin and were washed twice in buffer containing oxaliplatin at the same concentration as in the primary incubation mixture. Platelet-associated immunoglobulins were then detected by flow cytometry using fluorescein isothiocyanate-tagged anti-human IgG (Fcg-specific) and phycoerythrin-tagged anti-human IgM (Fcm-specific). Sera from normal, healthy donors, and sera containing previously identified quinine-dependent platelet antibodies served as negative and positive controls, respectively. A positive reaction was defined as a mean platelet fluorescence intensity at least twice that of platelets processed identically except for the absence of oxaliplatin. As shown in Fig. 4, patients' sera were incubated with normal group O platelets in the absence and presence of oxaliplatin. Both patients were confirmed to have oxaliplatin-dependent IgG platelet antibodies. In patient 1, a weak non-drug-dependent antibody was also present, and the significance of which is uncertain.

Drug-induced immune-mediated thrombocytopenia (DIIT) is diagnosed by demonstrating an antibody in the patient's serum that reacts with normal platelets in the presence of soluble drug [10]. Fewer than 10 chemotherapeutic agents have been shown to cause DIIT [3]. In patients receiving chemotherapy, thrombocytopenia is usually due to myelosuppression; however, acute thrombocytopenia of unknown cause should prompt suspicion for this entity. For DIIT, discontinuation of the offending drug is the most important treatment. Platelet transfusion is frequently needed when severe thrombocytopenia occurs with consequent mucocutaneous bleeding as in Patient 1. Whether corticosteroids are helpful is uncertain. Both our patients developed DIIT despite receiving dexamethasone as part of antiemetic regimen prior to oxaliplatin treatment.

Immune-mediated thrombocytopenia has been recognized as a rare adverse outcome of oxaliplatin infusion and has been identified in approximately 7% of allergic-type reactions to oxaliplatin in a retrospective analysis [11]. We have summarized all published case reports related to oxaliplatin-induced acute thrombocytopenia in table S1 (see additional file 1) [3,12-14] and table S2 (see additional file 2) [15-25], with table S2 reflecting reports without documentation of oxaliplatin-induced platelet antibodies. Each patient received prior oxaliplatin treatment, and all except one had metastatic CRC. Affected patients were predominately female. As shown in table S2 (additional file 2), acute thrombocytopenia with hemolysis (Evan's syndrome) has also been described in patients with hypersensitivity reaction from oxaliplatin. Furthermore, most patients with Evan's syndrome or hemolytic uremic syndrome likely from multiple exposures to oxaliplatin, presented with back pain during oxaliplatin infusion.

Taleghani et al. described oxaliplatin-induced immune pancytopenia four hours after the 15^th ^oxaliplatin treatment in a patient with advanced CRC [12]. They identified oxaliplatin-dependent antibodies to platelets, red blood cells and neutrophils. The patient developed significant pancytopenia upon re-treatment with oxaliplatin 30 minutes after infusion, with inception of hypersensitivity reaction. Curtis et al. reported two patients with metastatic CRC who developed acute thrombocytopenia one to two days after repeated (more than 10 times) oxaliplatin treatment [3]. Oxaliplatin-dependent antibodies specific for the platelet glycoprotein IIb/IIIa complex were identified in both patients' sera. As summarized in table S1 (additional file 1), primarily female patients were more likely to develop immune-mediated thrombocytopenia after repeated oxaliplatin treatment. Recovery from thrombocytopenia was observed in all cases after discontinuation of oxaliplatin. The majority of patients received platelet transfusion.

Moreover, as shown in tables S1 and S2 (additional files 1 and 2), some patients had received other chemotherapeutic agents such as irinotecan, cetuximab, bevacizumab and 5-FU after recovery from oxaliplatin-induced acute thrombocytopenia, and none of them had recurrent acute thrombocytopenia. This observation indicates that oxaliplatin-induced platelet antibodies do not cross-react with other chemotherapeutic agents. In our report, patient 1 resumed treatment three months later with 5-FU and LV, with later addition of bevacizumab. Patient 2 received bevacizumab and 5-FU, with addition of irinotecan. Neither patient experienced recurrent hypersensitivity reaction or recurrent episode of acute thrombocytopenia. Bozec et al. reported a case of a 53-year-old male with metastatic CRC, previously treated with FOLFOX, who presented with irinotecan-induced immune thrombocytopenia 18 hours after the onset of hypersensitivity reaction during the first irinotecan treatment [26]. The patient experienced another episode of hypersensitivity reaction followed by acute thrombocytopenia during the second irinotecan infusion. IgG platelet antibody in the presence of irinotecan was identified in the serum. Since the patient developed immune thrombocytopenia during the first irinotecan infusion, the authors speculated that this patient's drug-dependent platelet antibody could have been prompted by the previously administered chemotherapeutic agent which appeared to possess the ability to cross-react with irinotecan. It is likely that oxaliplatin might have been the culprit in their case; however, analysis for the presence of oxaliplatin-dependent platelet antibody was not performed to support this hypothesis.

Our report highlights the importance of promptly recognizing the association between oxaliplatin-induced hypersensitivity reaction and immune-mediated thrombocytopenia. Female patients with advanced CRC and prior oxaliplatin exposure are more likely to develop this consequence, although it may also occur in male patients. Therefore, it is imperative that patients should be thoroughly examined for signs and symptoms of bleeding, with concurrent CBC evaluation even after recovery from the hypersensitivity symptoms. Heightened vigilance and prompt testing may be helpful in recognizing development of antibody in patients with religious objections to transfusion before the onset of severe thrombocytopenia. Patients usually respond to discontinuation of oxaliplatin, with concurrent platelet transfusion if indicated. Additional tests such as peripheral blood smear examination and direct antiglobulin test are also helpful in assessing Evan's syndrome or hemolytic-uremic syndrome if worsening anemia or hemolysis is noted. Patients with documented oxaliplatin-induced acute thrombocytopenia should not be re-challenged with oxaliplatin and should have oxaliplatin listed as a drug allergy.

## Competing interests

The authors declare that they have no competing interests.

## Authors' contributions

The two case reports were originated by MAB, WTS, CSC and CTH. BRC and RHA carried out the assays for oxaliplatin-dependent platelet antibody. All authors participated in drafting and editing the manuscript. All authors read and approved the final manuscript.

## Consent

Written informed consent was obtained from each patient for publication of this case report and accompanying images. A copy of the written consent is available for review by the Editor-in-Chief of this journal.

## Supplementary Material

Additional file 1 **Table S1. Oxaliplatin-induced immune-mediated thrombocytopenia**. Summary of all published case reports related to oxaliplatin-induced acute thrombocytopenia with documentation of oxaliplatin-induced platelet antibodies. Click here for file

Additional file 2 **Table S2. Other oxaliplatin-related acute thrombocytopenia reports**. Summary of all published case reports related to oxaliplatin-induced acute thrombocytopenia without documentation of oxaliplatin-induced platelet antibodies. Click here for file
