# Supplementary material for: Hypersensitivity reaction and acute immune-mediated thrombocytopenia from oxaliplatin: two case reports and a review of the literature
Source: J Hematol Oncol. 2010 Mar 26;3:12. doi: 10.1186/1756-8722-3-12 (PMC2859393; doi:10.1186/1756-8722-3-12)
Supplement: Additional file 1 — Table S1. Oxaliplatin-induced immune-mediated thrombocytopenia. Summary of all published case reports related to oxaliplatin-induced acute thrombocytopenia with documentation of oxaliplatin-induced platelet antibodies. [file 1756-8722-3-12-S1.DOC]

| Table 1. Oxaliplatin-induced immune-mediated thrombocytopenia | | | | | | | | |
| --- | --- | --- | --- | --- | --- | --- | --- | --- |
| Author | Infusion number of oxaliplatin | Age/gender | Onset of thrombocytopenia and/or bleeding after oxaliplatin | Hypersensitivity reaction from oxaliplatin | Nadir of platelet count (/µL) | Oxaliplatin-dependent platelet antibodies | Treatment | Outcome/ subsequent treatment |
| Taleghani et al. [12] | 15 and 17 | 79/F | 4 h (15^th^); within 30 min of oxaliplatin infusion (17^th^) | No (15^th^); yes (17^th^) | 5,000 (15^th^)/ 7,000 (17^th^) | GP IIb/IIIa, Ia/IIa, Ib/Ix; antibodies against WBC/RBC noted | Transfusion and steroid | Recovery |
| Curtis et al. [3] | 17 | 38/F | 2 d | No | 6,000 | GP IIb/IIIa | Transfusion | Recovery/ irinotecan |
| Curtis et al. [3] | 10 and 11 | 55/F | 24 h (10^th^); 4 h (11^th^) | No | 6,000 (10^th^) / 2,100 (11^th^) | GP IIb/IIIa | Transfusion | Recovery |
| Pavic et al. [13] | 20 | 59/F | 8 h | No | 5,000 | GP IIb/IIIa, Ia/IIa, Ib/Ix | Steroid | Recovery |
| James et al. [14] | 28 | 60/F | 7.5 h | No | 0 | IgG | Transfusion and steroid | Recovery/ bevacizumab, 5-FU, LV and irinotecan |
| Table 1. Oxaliplatin-induced immune-mediated thrombocytopenia (continued) | | | | | | | | |
| Bautista et al. 2010 | 3 (2^nd^ course) | 60/M | Immediately | Yes | 4,000 | IgG | Transfusion | Recovery/ observation for 3 months |
| Bautista et al. 2010 | 3 (2^nd^ course) | 66/F | Within 10 min of oxaliplatin infusion | Yes | 66,000 | IgG | Steroid | Recovery/ bevacizumab, 5-FU and irinotecan |

M: male; F: female; GP: glycoprotein; IgG: immunoglobin G; 5-FU: 5-fluorouracil; LV: leucovorin
