# Supplementary material for: Hypersensitivity reaction and acute immune-mediated thrombocytopenia from oxaliplatin: two case reports and a review of the literature
Source: J Hematol Oncol. 2010 Mar 26;3:12. doi: 10.1186/1756-8722-3-12 (PMC2859393; doi:10.1186/1756-8722-3-12)
Supplement: Additional file 2 — Table S2. Other oxaliplatin-related acute thrombocytopenia reports. Summary of all published case reports related to oxaliplatin-induced acute thrombocytopenia without documentation of oxaliplatin-induced platelet antibodies. [file 1756-8722-3-12-S2.DOC]

| Table 2. Other oxaliplatin-related acute thrombocytopenia reports | | | | | | | | | |
| --- | --- | --- | --- | --- | --- | --- | --- | --- | --- |
| Author | Infusion number of oxaliplatin | Age/gender | | Onset of thrombocytop-enia and/or bleeding after oxaliplatin | Hypersensitivity reaction from oxaliplatin | Nadir of platelet count (/µL) | Antibodies | Treatment | Outcome/  subsequent treatment |
| Acute thrombocytopenia | | | | | | | | | |
| Dold et al. [15] | 17, 19 (no event on 18) | 51/F | | 24 h (17^th^); 4 h (19^th^) | No (17^th^); yes (19^th^) | 7,000 (17^th^); 2,000 (19^th^) | Not done | Transfusion | Recovery |
| Beg et al. [16] | 5 (2^nd^ course) | 74/F | | During infusion | No | 6,000 | Platelet IgG and IgM antibodies | Transfusion | Recovery/  cetuximab and irinotecan |
| Beg et al. [16] | 2 (2^nd^ course) | 83/F | | 1 h after starting infusion | No, but altered mental status, nausea and diarrhea during infusion | 41,000 | Not done | Transfusion | Recovery/ bevacizumab, 5-FU, and LV |
| Acute thrombocytopenia with hemolysis (Evan’s syndrome) | | | | | | | | | |
| Sorbye et al. [17] | 9 | 52/M | | 1 d | No, but back pain during infusion and fever after each oxaliplatin | 17,000 | Positive DAT; no platelet antibodies | Transfusion | Recovery |
| Sorbye et al. [17] | 13 | 40/F | | 1 d | No, but back pain during infusion and fever after each oxaliplatin | 80,000 | Positive DAT; no platelet antibodies | No | Recovery |
| Earle et al. [18] | >15 | 56/M | | 5 h | No, but feeling unwell | 6,000 | Positive DAT | Steroid, transfusion | Recovery |
| Table 2. Other oxaliplatin-related acute thrombocytopenia reports (continued) | | | | | | | | | |
| Acute thrombocytopenia with hemolysis (Evan’s syndrome) (continued) | | | | | | | | | |
| Koutras et al. [19] | 14 | 60/M | | 1 h | Yes with fever, chills,  vomiting, abdominal and back pain. | 6,000 | Positive DAT | Transfusion and steroid | Recovery |
| Buti et al. [20] | 11 | 64/M | | Several hours | Yes with chills and back pain | 11,000 | Positive DAT; no platelet antibodies | Plasmapheresisand steroid | Recovery |
| Cobo et al.  [21] | 3 (2^nd^ course) | 59/F | | 24 h | No, but back pain during infusion | 7,000 | Positive DAT | Transfusion and steroid | Recovery |
| Santodir-occo et al. [22] | 15 | 44/F | | 1 h into infusion | Yes with chills, abdominal pain and nausea 1 h into infusion | 10,000 | Positive DAT; no platelet antibodies | Transfusion and steroid | Recovery |
| Shao et al. [23] | 24, | 64/M | | 1 d | No, but noted during prior infusion; back pain during infusion | 4,000 | Not done | Transfusion | Death due to intracranial hemorrhage |
| Acute thrombocytopenia with hemolytic-uremic syndrome | | | | | | | | | |
| Dahabreh et al. [24] | 4 on adjuvant treatment | | 52/M | Immediately with gross hematuria | No | 50,000 | Negative DAT and IAT | Steroid, fresh frozen plasma, hydration and diuretics | Recovery |
| Phan et al. [25] | 5 | | 65/M | Gross hematuria shortly after infusion | No, but back pain during infusion | 24,000 | Negative DAT; positive platelet antibodies | Transfusion and hemodialysis | Recovery/ irinotecan, 5-FU and LV |

M: male; F: female; DAT: direct antiglobulin test; IAT: indirect antiglobulin test; 5-FU: 5-fluorouracil; LV: leucovorin
